# Assessment of dietary intake and body composition of participants in the LatinAmerican Initiative for Lifestyle Intervention to Prevent Cognitive Decline ‐ LatAm‐FINGERS

**DOI:** 10.1002/alz70860_105875

**Published:** 2025-12-23

**Authors:** Jessica Abdo Goncalves Tosatti, Vitoria Silva Vieira, Andre Luis Lopes, Jessica Diniz Pereira, Lais Soares Figueiredo, Dalila Maria Dutra Lima, Karina Braga Gomes, Maira Tonidandel Barbosa, Paulo Caramelli

**Affiliations:** ^1^ Faculty of Medicine ‐ Federal University of Minas Gerais, Belo Horizonte, Minas Gerais, Brazil; ^2^ Nursing School ‐ Federal University of Minas Gerais, Belo Horizonte, Minas Gerais, Brazil; ^3^ School of Physical Education, Physiotherapy and Occupational Therapy ‐ Federal University of Minas Gerais, Belo Horizonte, Minas Gerais, Brazil; ^4^ Faculty of Pharmacy ‐ Federal University of Minas Gerais, Belo Horizonte, Minas Gerais, Brazil

## Abstract

**Background:**

The Latin American Initiative for Lifestyle Intervention to Prevent Cognitive Decline (LatAm‐FINGERS) is the first multicenter, non‐pharmacological randomized clinical trial in the field of dementia developed in Latin America. The study aims to investigate the influence of a multidomain intervention on cognitive impairment in older adults with associated risk factors, including cognitive training, physical activity, clinical monitoring and changes in eating habits.

**Method:**

Participants underwent dietary habit change interventions conducted by a nutrition team. Monthly, group discussions focused on topics presented in the “Food Guide for the Brazilian Population”. After 12 months of intervention, individualized consultations with a nutrition team were conducted to assess food intake using a 24‐hour recall method, obtaining data on the consumption of macronutrients, fiber, calcium, vitamins D and B12, and body composition based on weight and height measurements, as well as anthropometric measurements. The data were tabulated, and the results were presented as mean and standard deviation or relative frequency.

**Result:**

Twenty‐two participants from the systematic group were evaluated, with a mean age of 69.4 ± 4.9 years, predominantly composed of women (54.5%). Regarding the assessment of food consumption, the average water intake was 1430 ± 529 ml, the average caloric intake was 1568.3 ± 501.3 calories, carbohydrate intake was 176.2 ± 64.8 grams, protein intake was 77.3 ± 37.9 grams, lipid intake was 60.9 ± 24.6 grams, and fiber intake was 18.0 ± 9.0 grams. In terms of micronutrients, the average calcium intake was 575.9 ± 267.7 mg, Vitamin D was 1.6 ± 2.1 μg, and Vitamin B12 was 1.9 ± 3.0 μg. For anthropometric markers, the mean weight of the participants was 71.4 ± 17.4 kg, body mass index (BMI) was 27.3 ± 5.4 kg/m^2^, neck circumference was 36.9 ± 4.0 cm, waist circumference was 90.7 ± 14.7 cm, arm circumference was 30.2 ± 4.3 cm, calf circumference was 37.3 ± 4.2 cm, waist‐to‐hip ratio was 0.9 ± 0.1, and waist‐to‐height ratio was 0.6 ± 0.1.

**Conclusion:**

The results highlight the need for personalized nutritional interventions for older adults to improve the intake of critical micronutrients and macronutrients to reduce metabolic risks.

